# A bibliometric analysis of the 50 most cited articles on acromioclavicular joint reconstruction

**DOI:** 10.1007/s12306-025-00886-w

**Published:** 2025-01-30

**Authors:** L. O’Dwyer, B. Murphy, M. S. Davey, D. Morrissey, J. T. Cassidy

**Affiliations:** 1 Graduate Entry Medical School, University of Limerick, Co. Limerick, Republic of Ireland; 2 Department of Trauma & Orthopaedic Surgery, Cork University Hospital, Wilton, Cork, Republic of Ireland; 3 Department of Trauma & Orthopaedic Surgery, University Hospital Limerick, Dooradoyle, Co. Limerick, Republic of Ireland

**Keywords:** ACJ reconstruction, Citation, Bibliometric, Orthopaedic surgery, Shoulder surgery

## Abstract

The aim is to identify the 50 most cited papers and thus the most influential papers pertaining to ACJ reconstruction, and specifically, analysing the level of evidence (LOE), article content, journals occurring, and countries represented within the 50 most cited. A search of the Web of Science database was carried out using the following terms: “Acromioclavicular joint” OR “AC joint” (Topic) AND Reconstruction OR Repair (Topic). The top 50 relevant articles were analysed in relation to citations, citation density, geographic origin of the article, year published, and article type. The articles were cited a total of 6053 times. The most cited article was cited 347 times. The highest citation density was 20.02, with a mean citation density of 7.71 ± 4.13. Seventy per cent of the articles involved clinical research, 74% of which involved level IV evidence. Fifty-two per cent of the articles were published in the *American Journal of Sports Medicine (AJSM).* Most authors originated from USA (n = 26 or 52%), followed by Germany (*n* = 14 or 28%). This study revealed a paucity of articles with higher LOE among the most cited. Eight of the top 10 are either theoretical or biomechanical studies, and one reports an examination technique. Only two of the top 10 reported outcomes following surgical intervention and can thus directly guide treatment. Future research in the area of ACJ reconstruction should focus on generating high-quality interventional studies capable of informing/impacting patient care. Publishing in journals such as *AJSM* or *Arthroscopy* may lead to more citations.

## Introduction

Acromioclavicular joint (ACJ) injuries are common, accounting for 40% of shoulder injuries following trauma [1]. The radiological assessment of ACJ separation is categorised by the Rockwood classification, which has a poor correlation with pain and function [2, 3]. There is no consensus regarding when/when not operative and, upon surgical intervention, the optimal mode of fixation [4–9]. A recent consensus statement from the ISAKOS group suggested that types 1–2 should be treated conservatively, while types 4–6 should be treated surgically [10]. Type 3 injuries are then separated into types 3A (stable) and 3B (unstable). Patients with type 3A injuries with no persisting pain and full function should be treated nonsurgically, while patients with type 3B injuries should be treated surgically.

The number of citations an article receives can be used to quantify its significance or impact [11]. Using these citations, journal editorial boards may generate their journal’s impact factor (IF), suggesting how citable studies published in their journal may be and therefore drawing the scientific community to read the journal’s content [12]. The calculation of the IF is based on two steps: (1) the numerator (number of citations in the current year from articles published in the previous two years) divided by (2) the denominator (number of articles published in the previous two years) [13].

Bibliometric analysis is a review methodology that allows for the tracking of article, journal and researcher impact while also uncovering emerging trends within the field. Previous bibliometric analyses have been performed in various orthopaedic fields, such as hip and knee arthroplasty [14], spinal deformity surgery [15], and foot and ankle surgery [16]. There are even more sub-specialist topics, such as the Ilizarov technique [17]. There are also studies pertaining to shoulder surgery [18] and shoulder arthroscopy [19], but to date, there has been no bibliometric study examining ACJ reconstruction.

The purpose of this study was to identify the 50 most cited papers and thus the most influential papers pertaining to ACJ reconstruction, and specifically, analysing the level of evidence (LOE), article content, journals occurring, and countries represented within the 50 most cited. The following is hypothesised: 1) the LOE among the top 50 will be poor; 2) there will be a variety of basic science and clinical studies among the list; and 3) there will be a select few “prestigious” journals among the most cited.

## Methods

### Search strategy

A bibliometric analysis of articles pertaining to ACJ reconstruction was performed. A search of the Web of Science database was conducted on the 11 July 2023. The Boolean search terms used were “Acromioclavicular joint” OR “AC joint” (Topic) AND Reconstruction OR Repair (Topic). The inclusion criteria were as follows: (1) published prior to 11 July 2023 and (2) related to ACJ reconstruction (including epidemiology, diagnosis, treatment options (operative and nonoperative), rehabilitation and postoperative care). The exclusion criteria were as follows: (1) no available English language translation; and (2) studies exclusively relating to clavicular fracture, shoulder impingement, or articles unrelated to ACJ reconstruction.

### Analysis

This resulted in a total of 874 articles returned. The search results were ordered by citation number (highest first). The title and abstract of each article were then checked for relevance. The 50 most cited studies relevant to ACJ reconstruction were downloaded for analysis, and a full-text review was carried out. An Excel file was created with the following details: (1) citations, (2) citation density, (3) authors, (4) title, (5) year of publication, (6) source journal of the article, (7) geographic origin of the authors, (8) article type (basic science article, clinical research article), and (9) the LOE for the clinical research article. The analysis was conducted using a combination of Excel and Web of Science Clarivate Analytics.

### Metric calculation

The citation density was calculated by dividing the number of citations an article had by the number of years that had passed since its publication. The LOE was determined using guidelines published by *The Journal of Bone and Joint Surgery* [20].

## Results

**Fig. 1:**
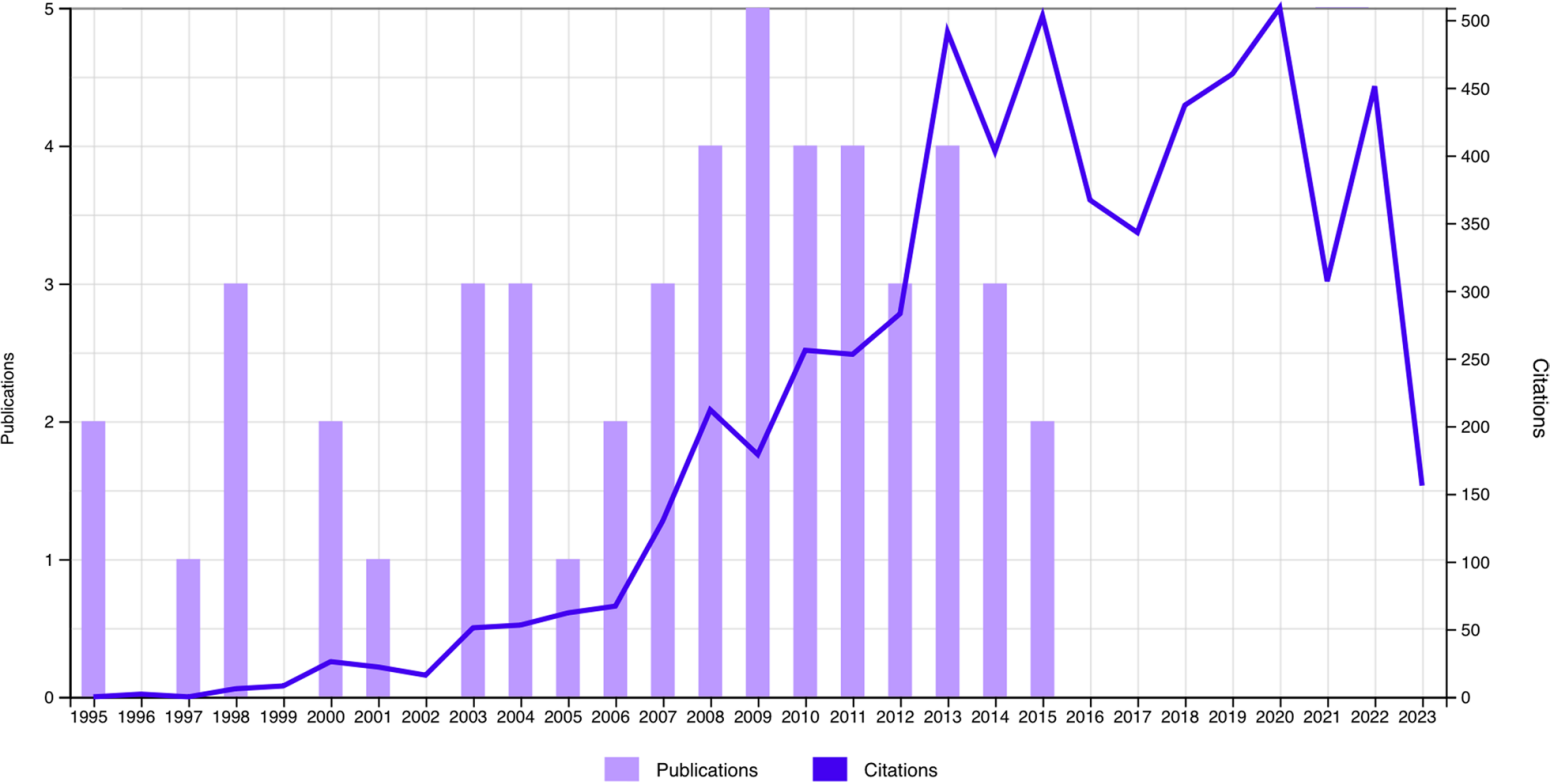
Graph shows the trend of publications and citations

The 50 most cited articles can be found in Table 1. These articles were cited a total of 6,053 times. On average, each article was cited 121.06 times. When excluding self-citations, the total number of citations was 5,767. The most cited article was O’Brien et al., which was published in 1998 [21] and was cited 347 times. The highest citation density was 20.09, which was a paper by Beitzel et al. published in 2013 [22]. The mean citation density for the 50 studies was 7.71 ± 4.13. The most cited article [21] dropped to fifth place in the citation density table. The publication year for the articles ranged from 1995 to 2015. The year with the greatest absolute number of publications was 2009, with five articles each, followed by 2008, 2009, 2010, 2011, and 2013, which produced four articles each. Only six of the articles in this list were published before 2000. The year with the greatest number of citations was 2020 (n = 509), followed by 2015 (n = 503) and 2013 (n = 491). These can be seen in the trend line in Fig. 1.

**Table 1:** Fifty most cited articles pertaining to acromioclavicular joint reconstruction

Total citations (Citation density)	Article title (year)	Authors
347 (13.35)	The active compression test: A new and effective test for diagnosing labral tears and acromioclavicular joint abnormality (1998)	O’Brien; Pagnani; Fealy; Mcglynn; Wilson
307 (18.06)	Evaluation and treatment of acromioclavicular joint injuries (2007)	Mazzocca; Arciero; Bicos
283 (15.72)	A biomechanical evaluation of an anatomical coracoclavicular ligament reconstruction (2006)	Mazzocca; Santangelo; Johnson; Rios; Dumonski; Arciero
242 (18.62)	Arthroscopically Assisted Stabilisation of Acute High-Grade Acromioclavicular Joint Separations (2011)	Scheibel; Droschel; Gerhardt; Kraus
221 (20.09)	Current Concepts in the Treatment of Acromioclavicular Joint Dislocations (2013)	Beitzel; Cote; Apostolakos; Solovyova; Judson; Ziegler; Edgar; Imhoff; Arciero; Mazzocca
221 (13.00)	Anatomy of the clavicle and coracoid process for reconstruction of the coracoclavicular ligaments (2007)	Rios; Arciero; Mazzocca
197 (12.31)	The Anatomic Reconstruction of Acromioclavicular Joint Dislocations Using 2 tightrope Devices A Biomechanical Study (2008)	Walz; Salzmann; Fabbro; Eichhorn; Imhoff
181 (9.05)	Biomechanical rationale for development of anatomical reconstructions of coracoclavicular ligaments after complete acromioclavicular joint dislocations (2004)	Costic; Labriola; Rodosky; Debski
170 (12.14)	Arthroscopically Assisted 2-Bundle Anatomical Reduction of Acute Acromioclavicular Joint Separations (2010)	Salzmann; Walz; Buchmann; Glabgly; Venjakob; Imhoff
160 (6.67)	Structural properties of the intact and the reconstructed coracoclavicular ligament complex (2000)	Harris; Wallace; Harper; Goldberg; Sonnabend; Walsh
153 (7.29)	All-arthroscopic versus mini-open rotator cuff repair: A long-term retrospective outcome comparison (2003)	Severud; Ruotolo; Abbott; Nottage
147 (7.74)	Anatomical acromioclavicular ligament reconstruction—A biomechanical comparison of reconstructive techniques of the acromioclavicular joint (2005)	Grutter; Petersen
141 (9.40)	Semitendinosus Tendon Graft Versus a Modified Weaver-Dunn Procedure for Acromioclavicular Joint Reconstruction in Chronic Cases A Prospective Comparative Study (2009)	Tauber; Gordon; Koller; Fox; Resch
136 (11.33)	Complications Related to Anatomic Reconstruction of the Coracoclavicular Ligaments (2012)	Milewski; Tompkins; Giugale; Carson; Miller; Diduch
135 (12.27)	Complications After Anatomic Fixation and Reconstruction of the Coracoclavicular Ligaments (2013)	Martetschlager; Horan; Warth; Millett
123 (10.25)	Epidemiology of Acromioclavicular Joint Injury in Young Athletes (2012)	Pallis; Cameron; Svoboda; Owens
119 (4.58)	The evaluation and treatment of the injured acromioclavicular joint in athletes (1998)	Lemos,
115 (7.67)	Acromioclavicular Joint Injuries: Diagnosis and Management (2009)	Simovitch; Sanders; Ozbaydar; Lavery; Warner
113 (7.53)	Incidence of Associated Injuries with Acute Acromioclavicular Joint Dislocations Types III Through V (2009)	Tischer; Salzmann; El-Azab; Vogt; Imhoff
113 (4.19)	Functional evaluation of the ligaments at the acromioclavicular joint during anteroposterior and superoinferior translation (1997)	Lee; Debski; Chen; Woo; Fu
110 (3.79)	Repair of complete acromioclavicular separations using the acromioclavicular-hook plate (1995)	Sim; schwarz; hocker; berzlanovich
104 (5.20)	Biomechanical function of surgical procedures for acromioclavicular joint dislocations (2004)	Jari; Costic; Rodosky; Debski
94 (9.40)	Has the arthroscopically assisted reduction of acute AC joint separations with the double tight rope technique advantages over the clavicular hook plate fixation? (2014)	Jensen; Katthagen; Alvarado; Lill; Voigt
94 (5.88)	Acromioclavicular dislocation Rockwood III-V: results of early versus delayed surgical treatment (2008)	Rolf; von Weyhern; Ewers; Boehm; Gohlke
92 (3.17)	Arthroscopically Assisted 2-Bundle Anatomic Reduction of Acute Acromioclavicular Joint Separations 58-Month Findings (2013)	Venjakob; Salzmann; Gabel; Buchmann; Walz; Spang; Vogt; Imhoff
92 (8.36)	Acromioclavicular separation—reconstruction using synthetic loop augmentation (1995)	Morrison; lemos
89 (5.56)	The Coracoidal Insertion of the Coracoclavicular Ligaments An Anatomic Study (2008)	Salzmann; Paul; Sandmann; Imhoff; Schottle
88 (3.83)	Arthroscopic reconstruction for acromioclavicular joint dislocation (2001)	Wolf; Pennington
87 (7.91)	Management of acute acromioclavicular joint dislocations: current concepts (2013)	Tauber,
84 (4.00)	Stability of acromioclavicular joint reconstruction—Biomechanical testing of various surgical techniques in a cadaveric model (2004)	Deshmukh; Wilson; Zilberfarb; Perlmutter
84 (4.20)	A modified technique of reconstruction for complete acromioclavicular dislocation—A prospective study (2003)	Tienen; Oyen; Eggen
80 (6.15)	Mid-term results after operative treatment of rockwood grade iii-v acromioclavicular joint dislocations with an ac-hook-plate (2011)	Kienast; thietje; queitsch; gille; schulz; meiners
80 (4.71)	Treatment of grade III acromioclavicular joint injuries—A systematic review (2007)	Spencer
79 (6.08)	Biomechanical Comparison of Arthroscopic Repairs for Acromioclavicular Joint Instability Suture Button Systems Without Biological Augmentation (2011)	Beitzel; Obopilwe; Chowaniec; Niver; Nowak; Hanypsiak; Guerra; Arciero; Mazzocca
78 (3.25)	Ligament mechanics during three degree-of-freedom motion at the acromioclavicular joint (2000)	Debski; Parsons; Fenwick; Vangura
77 (4.28)	Arthroscopic reconstruction of the acromioclavicular joint disruption: surgical technique and preliminary results (2006)	Chernchujit; Tischer; Imhoff
76 (5.43)	Value of additional acromioclavicular cerclage for horizontal stability in complete acromioclavicular separation: a biomechanical study (2015)	Saier; Venjakob; Minzlaff; Fohr; Lindell; Imhoff; Vogt; Braun
76 (8.44)	All-Arthroscopic Weaver-Dunn-Chuinard Procedure with Double-Button Fixation for Chronic Acromioclavicular Joint Dislocation (2010)	Boileau; Old; Gastaud; Brassart; Roussanne
75 (7.50)	Rotational and Translational Stability of Different Methods for Direct Acromioclavicular Ligament Repair in Anatomic Acromioclavicular Joint Reconstruction (2014)	Beitzel; Obopilwe; Apostolakos; Cote; Russell; Charette; Singh; Arciero; Imhoff; Mazzocca
75 (6.25)	The use of hook plate in type III and V acromio-clavicular Rockwood dislocations: Clinical and radiological midterm results and MRI evaluation in 42 patients (2012)	Di Francesco; Zoccali; Colafarina; Pizzoferrato; Flamini
75 (4.69)	Injuries to the acromioclavicular joint (2008)	Fraser-Moodie; Shortt; Robinson
73 (7.30)	Management of Acromioclavicular Joint Injuries (2014)	Li; Ma; Bedi; Dines; Altchek; Dines
73 (4.87)	Relative contribution of acromioclavicular joint capsule and coracoclavicular ligaments to acromioclavicular stability (2009)	Dawson; Adamson; Pink; Kornswiet; Lin; Shankwiler; Lee
73 (3.48)	Decision making: operative versus nonoperative treatment of acromioclavicular joint injuries (2003)	Bradley; Elkousy
73 (2.81)	Reconstruction of chronic and complete dislocations of the acromioclavicular joint (1998)	Guy; Wirth; Griffin; Rockwood
72 (8.00)	Complications After Arthroscopic Coracoclavicular Reconstruction Using a Single Adjustable-Loop-Length Suspensory Fixation Device in Acute Acromioclavicular Joint Dislocation (2015)	Shin, SJ; Kim, NK
72 (4.80)	Prevalence of concomitant intraarticular lesions in patients treated operatively for high-grade acromioclavicular joint separations (2009)	Pauly, S; Gerhardt, C; Haas, NP; Scheibel, M
69 (4.93)	Clinical Results of Single-Tunnel Coracoclavicular Ligament Reconstruction Using Autogenous Semitendinosus Tendon (2010)	Yoo; Ahn; Yoon; Yang
68 (4.86)	Dynamic Radio logic Evaluation of Horizontal Instability in Acute Acromioclavicular Joint Dislocations (2010)	Tauber; Koller; Hitzl; Resch
67 (5.15)	Acromioclavicular and coracoclavicular cerclage reconstruction for acute acromioclavicular joint dislocations (2011)	Ladermann; Grosclaude; Lubbeke; Christofilopoulos; Stern; Rod; Hoffmeyer

**Fig. 2:**
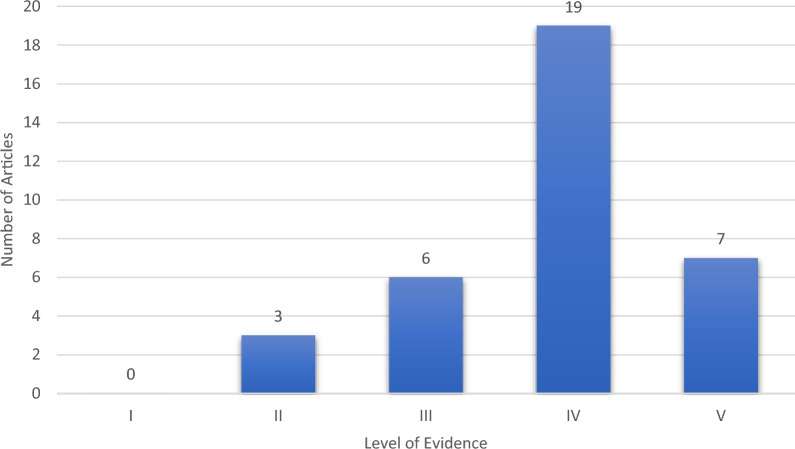
Number of articles by level of evidence (LOE)

The 50 studies were from 13 independent journals (Table 2). Fifty-two per cent of the articles were published in the *American Journal of Sports Medicine (AJSM)* (26/50). Seven journals had a single article feature in the top 50. The IF of the 13 journals ranged from 2.0 to 5.3 (Table 2), with a mean IF of 3.6 ± 1.0. The journal with the highest IF was the *Journal of Bone & Joint Surgery—American Volume (JBJS-Am Vol.)*. The journal with the lowest IF was *Clinics in Sports Medicine* (Fig. 2).

**Table 2:** Number of articles by source journal, with its impact factor

Journal title	Number of articles	Impact factor
American journal of sports medicine	26	4.8
Arthroscopy the journal of arthroscopic and related surgery	6	4.7
Archives of orthopaedic and trauma surgery	3	2.3
Clinical orthopaedics and related research	3	4.2
Knee surgery sports traumatology arthroscopy	3	3.8
Journal of shoulder and elbow surgery	2	3.0
Annals of biomedical engineering	1	3.8
Clinics in sports medicine	1	2.0
European journal of medical research	1	4.2
Injury international journal of the care of the injured	1	2.5
Journal of bone and joint surgery—American volume	1	5.3
Journal of bone and joint surgery – British volume	1	3.3
Journal of the American academy of orthopaedic surgeons	1	3.2

**Fig. 3:**
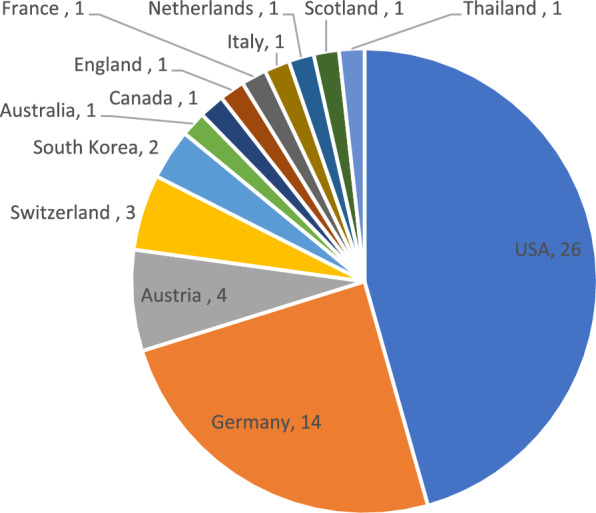
The number of articles by country of origin

Across the 50 articles, there were a total of 183 authors. The author who appeared most frequently was Imhoff AB, who appeared nine times. The authors originated from 13 countries (Fig. 3). Most authors originated from the USA (n = 26 or 52%), followed by Germany (n = 14 or 28%). Eight countries were represented just once. All the papers in this list were published in English.

The 50 articles consisted of 15 basic science articles (30%) and 35 clinical studies (70%). None of the articles on this list had level I evidence. The majority were level IV (n = 19) case series. There were three level II articles, six level III articles, and seven level V articles (Fig. 2). Almost three-quarters of the clinical research articles were level IV or less (74%). The basic science articles were primarily cadaveric studies testing the biomechanical properties of the ACJ. Only one of the basic science articles was published before 2000.

## Discussion

The most important findings of this review are as follows: (1) a study describing a clinical examination technique holds citation dominance, while a review on the current concepts in the evaluation and treatment of ACJ injuries has the highest citation density; (2) there is a paucity of high-quality studies among the most cited studies pertaining to ACJ reconstruction; (3) the *AJSM* is the most frequently occurring journal among the most cited articles; and (4) the USA and Germany are the most represented among the top 50.

The “classic” paper in this study was “The active compression test” by O’Brien et al., published in 1998 [21]. The eponymously named O’Brien’s test is still commonplace in clinical practice 25 years later. Its high number of absolute citations may be due to a phenomenon known as the “snowball effect” [18, 23], where authors cite an article because of previous citations. In terms of clinical relevance, acute ACJ separation can be diagnosed by clinical examination findings and can then be confirmed by imaging [24–26]. The O’Brien’s test remains an important step in the clinical evaluation of ACJ injury according to the ISAKOS group [10]. The article with the highest citation density was a systematic review by Bietzel et al. reporting on the current concepts in the treatment of ACJ dislocations [22]. This study aimed to clarify the following: (1) operative vs. nonoperative management, (2) early vs. delayed surgical intervention, and (3) anatomic vs. nonanatomic techniques. There is a lack of evidence to support treatment options for patients with ACJ separation. This study was limited by the lack of high-quality studies. Overall, this study demonstrated the value of review articles in informing both surgeon and patient populations on the management of ACJ injuries.

The LOE objectively grade’s the quality and, indirectly, the significance of a study. The majority of the articles in this top 50 list are level IV studies (19/50 or 38%), mostly therapeutic case series (12/50 or 24%). This is concerning because there is a paucity of high-quality research among the most cited articles pertaining to ACJ reconstruction. In contrast to a bibliometric analysis of the top 100 total hip arthroplasty studies, the LOE ranged from I to IV, with relatively high and even distribution [27]. This is an indication of the status of the available evidence for ACJ reconstruction. Only two of the top 10 studies on this list were interventional studies with outcomes measured [28, 29]. There were 14 additional interventional studies in the top 50. However, all but four of these are level IV interventional studies. Future studies should focus on producing high-quality interventional studies that can guide/impact clinical practice. However, studies deemed to have a lower LOE are still relevant as novel surgical techniques and treatment strategies are often presented as observational studies.

Exactly half of the articles in this study were published over a six-year period between 2008 and 2013. Twelve of the articles published at this time were case series related to various management approaches and surgical techniques for ACJ reconstruction. A popular era can indicate a time when there were major clinical advancements or paradigm shifts. Basic science studies from this era researched the biomechanical advantages of different approaches or techniques. These studies should springboard future high-level research in the area, such as high-quality RCTs and systematic reviews. Despite this, there has been no high-level evidence to support citation dominance within the field, and there is no consensus as to which therapeutic strategy or surgical technique is the gold standard.

Six studies published before 2000 were included in the top 50 list. The O’Brien study published in this era has been discussed above. Regarding the other studies, three were surgical technique case series [30–32], one was a review of the evaluation and treatment of ACJ injuries [33], and the last was a biomechanical study evaluating the ligaments in the ACJ [34]. Two of the case series described anatomic reduction techniques [30, 31], while the other described reconstruction using a hook plate [32]. Certain studies can lay the foundation for future research, with early biomechanical studies suggesting a move towards anatomic reduction [34] and early anatomic reduction techniques being refined throughout years of practice [30, 31]. Studies that may not reflect current best clinical practice or science can remain relevant if/when they continue to influence current research. For instance, the hook plate technique may be cited in current studies when discussing the range of techniques previously used [32]. If the study by O Brien et al. [22] is excluded, the highest citation density from the papers published before 2000 is 4.58, which is well behind the mean of 7.71. These studies may drop out of the 50 most cited lists in the years to come.

The majority (26/50 or 52%) of the articles in this study were published in *AJSM*. This journal featured heavily in similar studies focusing on shoulder surgery [18, 19]. The journal with the second most publications within this list was *Arthroscopy*. This also heavily featured in similar bibliometric studies [19, 20]. The journals that feature heavily on this list reflect their reputation within the orthopaedic community and are trusted to provide quality peer-reviewed research. These are the journals in which researchers seek to publish research while also providing a source for gaining knowledge. The *AJSM* has an IF of 4.8, which is the second highest in this study. Publishing in a high-impact journal does not necessarily imply higher quality, but the reputation of these journals ensures that their articles are read by a wider audience. Furthermore, recent large-volume review articles from such journals were not available for inclusion at the time of writing [35].

The *AJSM* is an American journal, and over half of the articles published originate from the USA (26/50 or 52%). Germany is also well represented (14/50 or 28%). This finding may suggest that authors publishing articles from other countries, where they do not have the same institutional resources, may be at a disadvantage. It may also suggest that these countries are leaders within this particular field and promote research and advancement within this area. The sheer number of articles published from these countries may enhance citation numbers, as authors may cite authors who are from a similar country of origin. The author who appeared most frequently was Imhoff AB, who originates from Germany. He appears in nine of these articles, which indicates a substantial contribution to the topic of ACJ reconstruction and to the progression of the area within his own country.

### Limitations

There are a number of limitations to this study. First, while trying to remain as objective as possible when deciding which of the articles to exclude from the initial search, there was an element of subjectivity when deciding which articles were relevant to ACJ reconstruction. To minimise this, the two first name authors reviewed the articles for relevance, and any articles for which there was ambiguity were then discussed with the senior authors. Furthermore, the absolute number of citations from an article is not a perfect measure of an article’s impact. This was adjusted by including citation density. There may have been articles with higher citation density not included in the top 50 list given that they were compiled as per absolute number. Importantly, the passage of time confers an advantage for overall citation number and means that recently published studies would not currently make this list, having not had much time to accrue citations. Therefore, an argument may be that it may be relevant to repeat this study in approximately 10 years to examine for changes; however, these data are current as possible possibly available at the time of writing. Finally, there are many areas of bias for which bias cannot be controlled, which can in turn increase the number of citations and citation density. Examples of this include self-citation by high-volume authors, citation of articles from journals in which they wish to publish, citation of articles that have been cited in previous articles on related topics, and citation by authors from the same country of origin.

## Conclusion

This study revealed a paucity of articles with higher LOE among the most cited articles. Eight of the top 10 are either theoretical or biomechanical studies, and one reports an examination technique. Only two of the top 10 reported outcomes follow surgical intervention and can thus directly guide treatment. Future research in the area of ACJ reconstruction should focus on generating high-quality interventional studies capable of informing/impacting patient care. Publishing in journals such as *AJSM* or *Arthroscopy* may lead to more citations.
